# Preventing the heterosexual epidemic: Responses and contingencies during the HIV/AIDS crisis in Denmark, 1981–1997

**DOI:** 10.1017/mdh.2025.10041

**Published:** 2026-01

**Authors:** Tobias de Fønss Wung-Sung

**Affiliations:** 1 University of Copenhagen Faculty of Humanities, Copenhagen, Denmark; 2 Museum Sønderjylland, Haderslev, Denmark

**Keywords:** HIV/AIDS, Denmark, Biomedical Politics, Gay Activism, Drug Use, Sex Work

## Abstract

HIV/AIDS posed a significant health threat in Denmark from the early 1980s until the end of the 1990s, claiming approximately 2,000 lives. Gay men, hemophiliacs, drug users, sex workers and migrants were overwhelmingly among the victims of the disease. They also constituted the groups most associated with it. This led to a raised level of public attention to these groups; a heightened visibility that ambiguously resulted both in improving the life conditions of some while also increasing the stigma of others. This article analyses the roles of different cross-sector actors in shaping the responses to HIV/AIDS in Denmark, with each group influencing and being influenced by the epidemic. Yet despite the clear connection between HIV/AIDS and the minoritized, often marginalized, groups, the article argues that the overarching and dominant response objective during the crisis in Denmark was to prevent a heterosexual epidemic. Throughout the crisis, other responses, aims and objectives concerning the groups most affected by HIV/AIDS could be, and did become, contingent with this dominant objective. The strengths and positions of those subresponses depended on, however, the perceptions of them as logical and tangible means to the primary end of preventing the heterosexual epidemic. Pulling together different and changing responses from different and changing actors serves to crystallize what objectives or logics are the mutual ones, and the significance of this analysis is that what appeared to be a very heterogeneous set of responses to the disease in Denmark, was in fact rooted in the same objective. Notably, the perceived pertinence of preventing the heterosexual epidemic was not rooted in actual rates of infection or spread of the disease.

## Introduction

On an autumn Sunday in 1989, Copenhagen City Hall in Denmark was the site of a worldwide first. Eleven same-sex, male couples signed documents which pronounced them registered partners, officially recognized by the Danish State, and guaranteed the rights of other married couples with only a few exceptions. First among them were Axel Axgil and partner Eigil, homophile pioneer and initiator in 1948 of the first openly gay and lesbian association in Denmark, Landsforeningen for Bøsser og Lesbiske (LBL). Forty-one years later, the association was not just celebrating the day; it was also the Danish authorities’ main civic partner in managing the HIV/AIDS crisis. Two years earlier, in 1987, Copenhagen was also where an unprecedented conference on drug use took place. Prominent guests, like the Ministers of the Interior and Social Affairs, sat alongside doctors, social workers, and activists discussing drug use in an atmosphere marked by the HIV/AIDS crisis. And in 1992 Copenhagen City Hall would once again house an unlikely meeting, this time about male sex work. At the same time, in the Danish Parliament, sex worker and activist, Jackie Siewens, was received as the guest of the Minister of Social Affairs at a meeting during which the two discussed the rights of sex workers in the face of the HIV/AIDS crisis.

First identified in the USA in 1981, the unknown disease later called AIDS spread, and its first victims in Denmark were diagnosed later that year. Attracting mostly the attention of medical professionals initially, the disease subsequently became a political and a civil society problem, in politics and in the affected communities. Due to international scientific invention, the disease was treatable by the late 1990s. From 1996, the so-called HAART therapy proved successful in preventing the HIV virus from causing AIDS. HIV/AIDS became a manageable yet still serious and stigmatized medical condition. While continuing to cause a severe health crisis across the Global South, claiming many more lives than it did in the Global North, around 2,000 individuals lost their lives to AIDS in Denmark. Many more contracted the HIV virus, or saw their lives marked by the disease in other ways. Besides the traumatic losses of many, often young, lives, HIV/AIDS also influenced the medical handling of and political engagement with diseases and health questions, including making more visible the groups hit hardest by it.

Such visibility was the reason why LBL’s role in responding to HIV/AIDS and the first registered partnership in 1989, the discussion of stances towards drug use and the subsequent easing of access to treatment, and the rights of sex workers becoming a political question during the HIV/AIDS crisis, were all related phenomena. The disease brought unlikely groups to the fore, and it contributed to shaping new alliances. Even if HIV/AIDS was met in some instances with silence, homophobia, and racism, subsequently leading to a backlash against gay rights and the further marginalization of other vulnerable groups, HIV/AIDS also catalyzed different kinds of change.^1^ The historian Christian McMillen has highlighted that a society’s understandings of infections becoming epidemics change with time: who is infected and how they become infected influence reactions and responses to epidemics.^2^ Recently, in connection with the COVID-19 pandemic, David Arnold et al. have argued that ‘pandemics function in ways that are more than simply corporeal or merely medical. They operate through words, and the meaning invested in neologisms and shifting metaphors’.^3^ In relation to understanding what epidemics do in societies, Jörg Vögele, Luisa Rittershaus, and Katharina Schuler suggest that they ‘act as catalysts for already existing developments and function as a stress test for the collective. In this way, societies can be analysed under epidemic crisis conditions as if under a magnifying glass and recurring patterns can be identified’.^4^

It has been argued that the HIV/AIDS crisis in Denmark was primarily understood as a homosexual problem, and that the country’s often lenient attitudes to homosexuality resulted in a similarly lenient stance on HIV/AIDS. In her comparison of HIV/AIDS in Denmark and Sweden, historian Signild Vallgårda argues that ‘cooperation and inclusion’ rather than ‘containment and control’ characterized the approaches and responses to HIV/AIDS in Denmark.^5^ Vallgårda maintains that HIV/AIDS in Denmark was understood politically as mostly ‘a homosexual problem’ while in Sweden, it was understood as one of drug use. Path dependencies in both countries, she shows – of a coercive stance on drug use in Sweden and a lenient approach to homosexuality in Denmark – shaped the nature of the responses in both countries. In the anthology ‘AIDS in the Industrialized Democracies’, political scientist Erik Albæk points in a similar vein to the comparative leniency of the Danish response to the disease. Albæk shows how an alliance of ‘medical professionals and various groups, most notably gay men’, constituted a political alliance whose objective was ‘the initiation of massive non-restrictive AIDS policies to combat the disease’.^6^ Recently, cultural studies scholars Camilla Bruun Eriksen and Michael Nebeling Petersen have also shown and argued that HIV/AIDS in Denmark not only gained signification as a homosexual problem but also as a racial one.^7^ As HIV/AIDS increasingly became associated with the racialized other, in particular with Asian female sex workers and black, African men, Danish responses to the disease developed along racialized lines.

These analyses are not wrong. In Denmark, HIV/AIDS overwhelmingly gained signification as a homosexual problem in the 1980s and as a racial one by the mid-1990s. The way in which the disease was handled in Denmark did also differ from neighbouring Sweden. This article will integrate, however, both analyses with a third one, connecting the two significations in time. Alongside being understood as a homosexual and a racialized problem, HIV/AIDS also gained signification as being – or not being – an issue for the heterosexual population. This signification applied across the significations of the disease as homosexual or racialized: It was not only identifiable in HIV/AIDS discourse at different times, but it was also the notion that became the catalyst of various responses to the disease throughout the epidemic. Even though the disease was mostly associated with minoritized, marginalized, and, to different degrees, stigmatized groups, the nexus of all responses to HIV/AIDS in Denmark was to prevent a heterosexual epidemic. Throughout the crisis, other responses, aims, and objectives concerning the groups most affected by HIV/AIDS could be and did become contingent with this dominant and primary objective. The strengths and positions of those sub-responses depended on, however, the perceptions of them as logical and tangible means to the primary end. Sub-responses included the advancement of improving the life conditions of gay and lesbians, as well as attention to solving issues related to drug users’ and sex workers’ marginalized positions. These agendas gained significance when perceived to prevent the heterosexual epidemic, but their significance diminished once that contingency disappeared. The identification of the heterosexual epidemic as the motor behind responses to the epidemic allows for two important methodological points: First, heterogeneous responses to diseases and epidemics in the same location do not necessarily need to be unrelated but can be anchored in the same objective or logic. Second, pulling together different and changing responses from different and changing actors can help crystallize what objectives or logics are the mutual ones. Notably, the perceived pertinence of such objectives and logics is not necessarily rooted in actual rates of infection or spread of the disease.

In this analysis, I pay attention to how the understandings of the disease, and the responses to it, changed over time. This approach takes inspiration from Paula Treichler’s argument that the AIDS epidemic is ‘simultaneously one of a transmissible lethal disease and an epidemic of meanings and signification’.^8^ Studying HIV/AIDS does not only mean having to navigate between social constructions of AIDS, for example, AIDS as a ‘gay plague’, or ‘a threat to civil rights’ and an ‘objective, scientific reality’.^9^ As Treichler points out, we also need to focus on ‘what we are told this [medical scientific] reality is: that is, on prior social constructions routinely produced within the discourses of biomedical science’.^10^ I study all the meanings and significations of HIV/AIDS as continuously constituted and negotiated. First, I discuss how understandings of the new disease emerged from medical and activist actors. Second, I examine how the activist-medical understanding of the HIV/AIDS crisis intersected with political responses. I then show how changing contexts influenced responses: who and how HIV/AIDS affected intersected with other understandings. Finally, I detect how the notion of the heterosexual epidemic diminished immediately prior to the medical breakthrough. I base my analyses and discussions on a diverse set of sources: on articles published in the largest medical journal in Denmark, *Ugeskrift for Læger* (UFL); on collections in the Danish National Archive from The National Association of Gays and Lesbians, *Landsforeningen for bøsser og lesbiske* (LBL), and The National Health Authority, *Sundhedsstyrelsen*; on publications and zines published by hemophiliac, gay, and sex working activists; and on digitized documents from the Danish Parliament, available on Folketingstidende.dk.

## The emergence of AIDS as a homosexual problem

Shortly after AIDS was first discovered in Denmark in 1981, it gained significance as a homosexual problem. The disease was mentioned in UFL for the first time in 1982, one article containing the descriptions of the first known four Danish cases: all homosexual men between the ages of 27 and 50.^11^ Another article asked ‘why this disease [had] come now?’, ‘whether it [was] about homosexuality?’ and ‘whether it [was] contagious?’ Meanwhile, the disease also obtained its meaning in the press. In the tabloid and broadsheet newspapers alike, the number of articles about AIDS increased in 1982, 1983, and 1984, before media attention to HIV/AIDS exploded in 1985. The disease became signified as a homosexual problem through articles with headlines in 1982 such as *Life-threatening cancer among homosexuals* ^12^ and *Homosexuals and drug addicts, get cancer from sniffing*,^13^ before eventually news stories turned more sensationalist and mortifying. By 1985, the tabloid *Ekstra Bladet* reported, for example, that ‘Every third gay man AIDS-infected’,^14^ ‘The gay community defy the AIDS-scare: We just continue fucking.’^15^

While the new disease became understood as a gay problem, the organized gay community refused to accept that only gay men could get AIDS. LBL wrote in its monthly magazine, PAN, that framing AIDS as a gay disease was a conspiracy and reactionary attempt of undermining the recent successes of the gay and lesbian movement, as well as a new weapon against gays and lesbians in the hands of the intolerant.^16^ Evidently, a new link between homosexuality and illness leading to unwanted public attention resonated all too clearly with the fact that the Danish Health Authority only removed homosexuality from its register of mental illnesses in 1981. Any attempt to link homosexuality with disease again was unacceptable for the organized gay activist community.^17^ On the contrary, and similar to homosexual organizations elsewhere,^18^ LBL was pursuing other goals. Undergoing a multifaceted reinvigoration since the mid-1970s, the association had purchased properties in both Aarhus and Copenhagen and launched new initiatives to improve the life conditions of homosexuals in Denmark.^19^

As the historian Craig Griffiths has shown in the case of West Germany, gay liberation activist efforts of transforming as well as integrating into mainstream society were characterized by an ambivalence that ran through the heart of the gay activist movement rather than one dividing it.^20^ Similarly, the gay activist landscape in Denmark included groups that rejected integration, even if such groups had lost their political momentums by the early 1980s.^21^ Some LBL activists perceived monogamy, marriage, and the nuclear family to be constraining consequences of capitalism. Instead of seeking inclusion, this faction worked for an independent homosexual subculture in which gays and lesbians could live free from shame and stigma. At the same time, LBL activists were also engaged in political lobbying for rights and inclusion.^22^ On the agenda for the 1980s, LBL’s political activists pursued a parliamentary appointment of a commission to scrutinize improvements to conditions for homosexuals in Denmark, including the implementation of anti-discrimination laws, labour market rights, and access to registered partnership.^23^ Notions of either position or, precisely, an ambivalence also characterized the Danish gay activists’ responses to the new disease. For the time being, however, and until the mid-1980s, LBL continued to ignore publically any particular connection between homosexuality and AIDS.

Among medical professionals, the understanding of the new disease as a homosexual problem arrived with the first notices of it. Medical communities in the USA reported an increase since 1981 of rare diseases in unlikely patient groups, in particular young people developing Kaposi’s sarcoma. US doctors understood the new illness as related to deficiencies of the immune system that only homosexual men seemed to develop. This directed their attention to the so-called homosexual lifestyle factors, in particular probing medical researchers to study whether the usage of poppers caused such immune deficiencies. In fact, the Cancer Institute in Aarhus had already launched in 1981 a study exploring the poppers thesis.^24^ Aspiring to examine what they believed to be a cohort of gay men with ‘different habits’ than gay men in the USA, the Aarhus researchers designed and distributed a questionnaire to be filled in by male members of LBL. The questionnaire inquired about lifestyle habits, including sexual practices and the usage of poppers.^25^ The researchers found that male members of LBL did not constitute a symptom-free cohort.^26^ AIDS was already in Denmark.

On the one hand, Danish medical professionals contributed to identifying AIDS as a homosexual disease. In 1983, Nils Strandberg Pedersen reported from his participation in a New Orleans conference that, ‘without understanding why, infections occurred in those with multiple sexual partners and in the receiving parties of inter-male anal intercourse’.^27^ On the other hand, the Danish doctors also quickly took notice of new groups suffering from AIDS, and Strandberg Pedersen also reported that the disease now appeared in drug-using and hemophiliac patients. Jan Gerstoft reported in another article, also from 1983, that anal sex in various forms was discussed as the issue and that the epidemiology of the new disease resembled that of Hepatitis B.^28^ He added that pursuit of the poppers thesis was over, and he concluded that:

We face an epidemic of a deadly, most likely contagious, disease. Hitherto, the disease has only hit specific and vulnerable patient groups, but there is no reason to believe that the disease will be confined in these groups in the future.^29^

Based on their participation in two 1983 conferences in the USA, Gerstoft and Strandberg Pedersen also presented suggestions as to how the new disease should be responded to. Homosexuals should be advised ‘to reduce their amount of sexual partners and ensure their partners [did] the same’,^30^ while ‘AIDS patients should not have any sexual contact with others’.^31^ In addition, Gerstoft and Strandberg Pedersen wrote that homosexuals should refrain from donating blood, while hemophiliacs should use ‘as little factor concentrate as possible’.^32^ Adding to these suggestions, two other epidemiologists, Viggo Faber and Jens Ole Nielsen, wrote in UFL in 1984, that ‘if AIDS [was] caused by a virus there must be degrees of infection’, including resistant patients with developed immunity upon infections.^33^ Nielsen supported the thesis that an unidentified virus caused AIDS, adding that:

It has been claimed that the AIDS epidemic has caused an epidemic of fear of the disease and an epidemic of homophobia. Fighting the latter two epidemics with proper information will hopefully provide the opportunity to rationally fight the first one.^34^

Analysed through the early articles in UFL, the Danish medical professionals were simultaneously attuned to the responses emerging from the USA, while they also considered how to circumvent emerging homophobia and fear. Other sources make clear that a group of Danish doctors, including Viggo Faber, were already familiar and in contact with LBL and its gay health activists. Such connections had taken shape during the years prior to AIDS, through a mutual interest in curtailing STDs among gay men. LBL health activist Tjerk van den Berg said, at an international Gay Health conference in Amsterdam in 1984, that LBL initiated a group in 1976, which established ties to the venereal clinics and a set of sympathetic doctors. He added that the ‘treatment of gay people [was] now better despite old conservative doctors who still hate[d] gay people’.^35^ By the beginning of 1980, the activists and the doctors launched new initiatives of anonymously collecting blood samples in gay saunas.^36^ Testing the samples, the doctors were able to conduct studies of venereal diseases among homosexual men,^37^ and the activists were able to prevent the registrations of sexual orientation they suspected took place.^38^

The existing connection between the doctors and the activists ensured the inclusion and influence of LBL on the Danish responses to AIDS. Faber initiated, for example, LBL chair Henning Jørgensen’s invitation to participate in the conference Aids in Europe – Status Quo 1983.^39^ According to a printed version of Jørgensen’s speech at the conference, LBL called for the establishment of venues for examination and treatment, and warned the authorities not to make gay men feel inferior.^40^ During the summer and autumn of 1984, Faber and leading doctors met with the LBL health activists again,^41^ where, according to the minutes, the medical professionals guaranteed the health activists examination anonymity and non-registration of sex partners. Furthermore, the doctors guaranteed that no ‘drastic measures would be applied’ and they accepted LBL’s suggestions of establishing the special clinics for examinations alongside special counselling groups. In return, the association promised to distribute the Danish Health Authority’s new information material targeted at gay men, printed in 8,000 copies. Transactions in which the authorities recognized LBL’s wishes for the handling of HIV/AIDS in exchange for the association’s voluntary work efforts later became characteristic of the relationship between the association and the authorities.

## Identifying the virus and the potential heterosexual epidemic

In Danish national politics, AIDS only slowly became a political problem. The first official recording of the new disease was in February 1984, when MP Jørgen Lenger asked the Minister of the Interior and responsible for health, Britta Schall Holberg, whether she would make permanent the AIDS examination clinics in the public health care system.^42^ The Minister confirmed, ^43^ adding tacitly that she acknowledged ‘the fear of this disease among certain vulnerable groups,’ and ending her response by stating that: ‘I would like to emphasize that generally, there are no reasons to fear AIDS.’^44^ Certain vulnerable groups referred here to homosexual men. Those who generally had no reason to fear AIDS referred, of course, to heterosexuals.

The year 1984 was also the year, however, when the international medical community established that infection with the HTLV-III virus, later HIV, caused AIDS, and by 1985, testing for viral antibodies became possible too.^45^ Able to dismiss that AIDS was only a male homosexual issue; medical, gay activist, and political responses all intensified in different ways. The medical authorities were alarmed by the epidemic potential of the new disease. While the absolute number of infections in Denmark remained low, prevalence increased, doubling by 1986 over a span of six to twelve months.^46^ With the new test results showing that between 24 and 28 percent of all tested homosexual men were antibody positive,^47^ LBL could no longer refrain from addressing the disease publically as a problem particularly – but not exclusively – for homosexuals.^48^ At the same time, however, it was a relief to LBL that not only homosexuals could get AIDS.

LBL’s pre-virus cooperation with the medical authorities led not only to an influence on the responses to AIDS, including concrete ways of handling testing anonymously, but also to the profiling of LBL as a trustworthy and appreciated partner of the authorities, to whom political actors were also prepared to listen. LBL succeeded in convincing the authorities that limiting the spread of infection in Denmark was contingent on improving the conditions for gays and lesbians. During a hearing at Denmark’s largest hospital, Rigshospitalet, in October 1985, for example, Henning Jørgensen said that HIV/AIDS risked setting back the improved conditions that gays and lesbians had experienced over the past 10–15 years, and that if those conditions were not further improved, the epidemic would simply spread.^49^ A year later, in 1986, LBL repeated the same argument. During a public debate, activist Jesper Jarmbæk explained that the HIV/AIDS strategy should depart from the fact that HIV/AIDS could be prevented only by implementing a change in behaviour through information about how to avoid risky sexual activities. LBL assured the authorities that the association would cooperate and that the best prevention against the spread of infection was a strong gay-lesbian community. LBL argued that the Danish AIDS strategy should also simultaneously combat discrimination against gays and lesbians and support the development of the homosexual subculture.^50^

The antibody test also revealed that a large number of haemophiliacs were already infected. A marginal group even smaller than the organized gay community, however, the Danish Association of Hemophiliacs, *Danmarks Bløderforening* (DBF), was also unable at first to attract political awareness sufficient to prevent virus transfusion through Factor-8 medication. That haemophiliacs were among the AIDS victims in the USA was known in Denmark from 1983, the year DBF also first responded to the news of the disease. DBF’s newsletter stated that the new disease was potentially blood-borne,^51^ although that concern seemed somewhat curtailed when drug users and homosexuals were shortly after banned from donating blood.^52^ DBF subsequently called for the National Health Authority to ensure Denmark becoming self-sufficient with blood supply and for all blood to be screened. DBF also wrote that heating blood might prove effective in reducing risk of contamination.^53^ At the same time, however, the newsletter stated that ‘unlike other risk groups, it [did] not look like haemophiliacs develop[ed] AIDS, even if they [had] developed anti-bodies against HTLV-III’,^54^ and that ‘fearing AIDS [should] not prevent anyone receiving Factor-8’.^55^

The uncertainty about what constituted a risk of contamination, and at what costs, delayed the political decisions about screening and heat-treating blood batches. Only in 1985, when the antibody test became fully available and test results showed that heat treatment of blood could remove antibodies, the government issued new policies. In addition, political attention to the haemophiliac problem and the blood-borne epidemic only intensified when the news reported of a traffic accident victim having received a blood transfusion with HIV-infected blood.^56^ By that time, 85 recipients of Factor-8 had tested positive^57^: A huge percentage of the estimated total number of only 300 haemophiliacs in Denmark. Even if only a small percentage of antibody positives were thought to eventually develop AIDS at this point, the new chair of DBF, Terkel Andersen, was right in his prediction in 1985 that ‘this [would] become the all-encompassing concern of the future for the association’.^58^ Soon after, DBF launched its first attempts of securing compensation for the infected patients and their families.^59^

The subsequent reactions to the delayed political intervention became a warning of how seriously the Danes took HIV/AIDS by 1985. The notion of a new virus causing a deadly disease scared the population, and the prospect of not only homosexual and haemophilic epidemics, but also a heterosexual one, only intensified that scare. Increasingly, the press began reporting that HIV/AIDS was an issue and disease for everybody. In April 1985, the broadsheet *Politiken* reported that a young boy in the USA had died after a blood transfusion.^60^ Although the numbers were later identified as exaggerated, *Politiken* also reported in the summer of 1985, on its front page, that 10,000 Danes were infected with the virus.^61^ In the same week, *Politiken* also quoted Viggo Faber, who said that ‘the number of victims’ would double in six months’.^62^ Such prognoses caused parts of Danish society to react in ways opposite to the response shaped by the medical-activist collaborations. In 1986, for example, the prison guard union demanded all prisoners in Denmark subjected to forced HIV testing.^63^ Some police wards called for a register of all known individuals with HIV, which one local ward actually compiled in practice.^64^ Also in 1986, one of the largest insurance companies in the country refused to issue life insurance to individuals with HIV, and in early 1987, a joint insurance industry planned to subject all known homosexual men, haemophiliacs, and drug users to HIV testing, providing they wanted to purchase insurance.^65^

The prospect of a heterosexual epidemic also meant that HIV/AIDS became a field of contestation for the government’s opposition. In Parliament, the left-wing opposition criticized Minister Britta Schall-Holberg for her lack of investment in preventing the factor-8 and blood transfusion crisis and for not securing the necessary means against the virus spreading among homosexual men.^66^ The left-wing opposition aligned with the positions of LBL and its AIDS activist group. LBL frequently received letters from sympathetic Danish MPs, offering the association to pose questions in Parliament,^67^ in particular from MP Jørgen Lenger from the left-wing party, Venstresocialisterne. Lenger actively positioned himself in the parliamentary discussions of the disease, highlighting the importance of cooperation with LBL and perpetuating the association’s argument that registration and compromising anonymity would be counterproductive.^68^ This illustrated that the gay activists had good allies in central positions – not only among medical professionals.

The right-wing opposition deployed a different strategy, proposing new legislation for the classification of HIV/AIDS as a venereal disease.^69^ This would have enabled the authorities to not only register cases but also subject citizens to forced testing and ultimately incarceration. Originating from the post-Second World War boom in venereal diseases, the laws had not been enforced for decades. The prospect of them being applied to HIV/AIDS sparked serious objections from not only gays and lesbians in LBL but also from haemophiliacs in DBF.^70^ Parliament rejected the first attempt in 1985, and the second in 1988 when the same party, *Fremskridtspartiet*, posed an identical proposal. The right-wing strategy eventually backfired, when a left-wing party raised a counter proposal of removing the venereal act altogether, and the House approved that in 1988.^71^

Meanwhile, political responses to HIV/AIDS were also taking shape in the National Health Authority, Sundhedsstyrelsen. The Health Authority’s principal task, to organize information about HIV/AIDS, required attention to different agendas in the medical, activist, and political arenas. In 1983, The Authority had drafted its first information leaflet about the disease. The Health Authority re-drafted it in 1984 and twice in 1985, as knowledge developed, and in accordance with the desired changes, especially presented by LBL.^72^ The Authority removed from its second 1985 publication, for example, its only recommendation in 1983 and 1984 of reducing the number of sex partners.^73^ Henceforth, the Health Authority did not refer to any notions of gay promiscuity as a risk. On the contrary, and in accordance with the wishes of LBL, the Authority encouraged only the practice of safer sex by condom usage for all, regardless of the number of partners.^74^

Some doctors supported the notion of changing individual behaviour in order to avoid infection while others objected to it. Notably, Dr. Jens Ole Nielsen was in favour of the approach. In 1986, he wrote in UFL that ‘to think legislation [could] change an epidemic of a sexually transferable disease [was] completely unrealistic and [lacked] historical information’.^75^ Others maintained that ‘the risk groups [should] reduce their number of sex partners … [and that] introducing the registration of LAV/HTLV-III positive individuals [was] necessary’.^76^ The different medical positions on AIDS thus reflected the different political responses; one faction in favour of more coercive measures and another attuned to the non-discriminatory aspirations, and the reliance on voluntary patient-authority cooperation. The Health Authority chose to align itself with the latter. All medical professionals agreed by 1985–1986, however, that the HIV/AIDS crisis was indeed a crisis. In UFL, several articles highlighted, for example, the alarming percentages of positive antibody tests among homosexual men,^77^ and that the number of AIDS patients grew exponentially.^78^ Jens Ole Nielsen wrote that ‘the objective epidemiological numbers [were] horrifying and that in no way [could] the spread of infection be said to be under control.^79^

From that point, the development of a much more comprehensive response to HIV/AIDS in Denmark took shape. The Health Authority set up an independent AIDS secretariat, headed by doctors Lone de Neergaard and Michael von Magnus, under which civil servants and an advisory group drew up the plans of a national safer sex campaign.^80^ The campaign was to be executed in close cooperation with the Danish regional councils and civic organizations, in particular LBL whose activists met in person with the new minister responsible for health, Knud Enggaard, in 1986.^81^ LBL expressed appreciation of the authorities’ accommodations of the association’s positions, and the minister agreed to allocate more funds to a campaign, directed specifically at gay men, called STOP AIDS. In addition, he agreed to ‘avoiding a Swedish model’,^82^ meaning one, compromising LBL’s demands of voluntariness and anonymity, areas in which the two neighbouring Scandinavian countries, Denmark and Sweden, navigated and handled the crisis in opposite ways.^83^ At the same time, LBL ensured the minister of its ability to enter into a partnership with the authorities. The association’s influence on both the development and the carrying out of the official Danish responses to HIV/AIDS was a reality. LBL was able to secure that AIDS telephone hotlines were set up in its premises in Copenhagen, Aarhus, and Odense. First initiated in Odense in 1985, the establishment of all three hotlines became a testimony to the trust that the authorities showed LBL. The hotlines provided information to all callers regardless of sexuality. In the agreement between the authorities and LBL, the minister insisted that the service should be relevant for all Danes, although the nature of the information was to be, as LBL preferred it, in a direct tone, free from moral stigma and judgment.^84^

## The changing epicentre of the heterosexual epidemic

In March 1987, a parliamentary resolution finally spelled out the principles of the official political response to HIV/AIDS in Denmark. The parties behind it agreed to ‘voluntariness, anonymity, honest and direct information’ being the ‘fundamental principles’ of the Danish response to AIDS, in order to ‘enable everyone’s confidence in the authorities and avoid all kinds of discrimination’.^85^ On paper, the resolution appeared as if drawn up by LBL’s activists, yet in the debate about it, more positions came to the fore. A number of politicians heralded LBL’s efforts thus far and accepted the position that HIV/AIDS posed a risk of setting back the improved life conditions of gays and lesbians in Denmark.^86^ At the same time, concerns about the spread of the epidemic in the heterosexual population were clear.

In particular, two members of parliament, MP for the centre-right party, Centrumdemokraterne, Birgith Mogensen, and MP for the right-wing party Fremskridtspartiet, Pia Kjærsgaard, expressed their concerns. Mogensen argued that ‘it [was] [her] impression that gay men practiced a form of self-regulation’^87^ but that such regulation did not apply to sex workers and drug users. She argued that ‘rent boys, prostitutes and drug addicts, however, [required] more outreach, [as] none of these groups [led] a responsible life’.^88^ On the topic of male sex workers, she claimed that:

It is a known fact that a large number of heterosexual young men practice homosexual prostitution for financial reasons. These so-called rent boys constitute the most dangerous AIDS contagion group. These young men have several sexual relations daily. They go from one man to the next. It is also a fact that they have heterosexual relationships. Our AIDS prevention work does not address this group, the most dangerous one. I believe that special outreach work is urgently needed here. This group should he tested and if they are HIV infected, it would be purposeful to criminalize those who continue their activities.^89^

In the same debate, Kjærsgaard added:

Drug addicts should have a very high priority, when we discuss initiatives, because so many drug addicts make their money in prostitution. This passes on the virus to the heterosexual part of the population and constitutes a time bomb under our future society. Perhaps this can lead to the prostitutes becoming subject to medical control. At least this would reduce the risk of contagion to that area.^90^

Notions of how the disease was understood differently, depending on who developed it and what this could potentially mean for society, were clear. Hemophiliacs were completely absent from the debate, and gay men were labelled responsible in contrast to sex workers and drug users. The medical, political, and activist responses were negotiated continuously in tandem with developing significations of HIV/AIDS. In that sense, the meanings of HIV/AIDS changed and responses adapted to different contexts. Homosexual men continued to constitute the majority of persons infected with HIV and dying of AIDS, but the homosexual epidemic did not develop as quickly and extensively as the medical authorities had feared. More importantly, the high infection prevalence did not transfer to the heterosexual population, and the medical and political responses to HIV/AIDS began focusing increasingly on drug use and sex work. While these groups never suffered the same infection and death rates, the disease was increasingly associated with them because they were seen as constituting the conduits of heterosexual infection.

Only one week prior to parliament passing the resolution, politicians and medical professional actors discussed the topic of drug users and HIV/AIDS at a meeting in Copenhagen. Even though the discussions brought forward echoes of Mogensen and Kjærsgaard’s concerns, the interventions suggested were less draconian than the MPs’ suggestions of medical control and criminalization. Head of the Danish County Organisation, *Amtsrådsforeningen*, Arne Grünfeld, argued, for example, that ‘there [was] no time for dogmatic positions and that open-mindedness [appeared] to be the way forward’.^91^ At the same time, however, he expressed a strong sense of urgency, asking, ‘how can we explain in the future, if we do not do the necessary now and the disaster strikes in 4-5 years?’^92^ Grünfeld’s dogmatic positions referred to the relationship between drug users and the authorities. Chair of the Danish Alcohol and Narcotics Council, Ingrid Langkilde, elaborated that less than half of all drug users were in contact with the health authorities, and she called for ‘new treatment options which drug users actually [wished] to make use of’, and to ‘meeting the individual where they [were]’.^93^

Dogmatic positions also referred to the use of methadone in the treatment of drug addiction – a practice falling out of favour in Denmark since the late 1970s.^94^ Minister Engaard argued for easing access to methadone and ‘adopting new approaches’ to drug users. Furthermore, he and another government minister present at the seminar voiced their support of the experimental distribution of free syringes in Copenhagen from pharmacies and a vending machine for off-hours close to the central station, an area known for hard drug usage. In addition, doctors from Funen and Jutland echoed the need to ‘surrender the idea of saving’ the drug users and ‘rather create a system based on their needs’.^95^ One doctor from Copenhagen, Joachim Knop, remained wary of methadone and argued that: ‘it limits AIDS contagion, yes, but it keeps drug addicts in abuse and is highly addictive’.^96^

Whereas the March seminar focused solely on drug use and HIV/AIDS and not on sex work, the AIDS Secretariat’s newsletter frequently addressed the two groups as one. In May 1987, the newsletter wrote that ‘IV drug addicts must be assumed to pose the greatest risk of the spread of AIDS to the heterosexual population by the usage of infected syringes and by sexual contact, in which drug using sex workers are a problem’.^97^ Later in 1987, the newsletter reported that ‘the drug problem [was] now central’, as there was a risk that drug users got the disease by sharing syringes and that ‘the virus [was] transferred from the drug users to the “regular”[sic.] heterosexual milieu’.^98^ The newsletter also reported that most politicians and authorities now favoured increased use of methadone and as well as access to social support.^99^ Ultimately, by the spring of 1988, the Alcohol and Narcotics Council changed its guidelines for methadone treatments, making it easier and faster for users to access methadone,^100^ and by 1989, over one million free syringes had been distributed in Copenhagen since the launch of the experiment in 1986.^101^

The Danish medical professionals never established that sex work contributed to the transmission of HIV/AIDS to the general population. Nevertheless, fantasies of the irresponsible and default-infected sex worker spreading the virus existed widely. Such fantasies caused further stigma and animosity against people in sex work. At the same time, however, the fantasy of HIV/AIDS spreading through sex work also brought attention to the phenomenon of selling sex and its consequences, even if the phenomenon of buying sex remained undiscussed. As was the case with the gay and lesbian community’s wishes for improved conditions, and for drug users’ access to methadone and clean syringes, HIV/AIDS also became a conduit for increased awareness about sex work in Denmark. New alliances between medical, political, and activist actors took shape, combining and realizing different objectives related to sex work.

The interconnections between HIV/AIDS and sex work were neither confined to Denmark nor only noticeable from a historical distance. As the crisis still unfolded, scholars, commentators, and activists in Denmark and beyond discussed how HIV/AIDS revitalized sex work stigma. Internationally, Graham Scambler and co-authors wrote in their article *Women prostitutes in the AIDS era*, for example, that by the time HIV/AIDS was ‘no longer framed as a gay disease, other groups became candidates for scapegoating’.^102^ The authors quoted other scholars who argued that AIDS was ‘medicalized as disease and moralized as stigma,’ and that ‘AIDS gave moralists another reason to condemn prostitution as morally wrong’. Scholars and commentators pointed to the historical precedence of women being seen as responsible for spreading venereal diseases to men, arguing that ‘women were seen as casual agents, rather than victims, of the spread of HIV’.^103^ Historians Marion Pluskota and Susan P. Conner have studied histories of prostitution in Amsterdam and Paris respectably. Pluskota argues that ‘the AIDS epidemic began simultaneously with an upsurge in hard drugs’, constituting a serious health threat to women selling sex. Subsequently, the municipality in Amsterdam initiated campaigns to promote the use of both condoms and clean needles.^104^ In Paris, the HIV/AIDS crisis hit a decade after sex workers unsuccessfully demonstrated for health benefits and reforms of the penal code in 1975–1976, but succeeded in becoming included in the public debates about sex work. Connor argues that sex workers formed a new social movement, gaining their own agency, in contrast to being excluded as ‘mere pawns’.^105^

In Denmark, similar questions of both stigma and blame – agency and action were also at stake. Jackie Siewens, a sex worker since her youth, published a magazine *Stå Sammen*, 1992–1995, with money she received from the AIDS Foundation. Her motivations were to unite sex workers in several ways. First, to unionize and demand legal changes, ultimately the abolition of the penal code, partially criminalizing commercial sex. Second, to protect sex workers against the threat of HIV/AIDS, against violence from sex buyers, and against discrimination from authorities and society. Third, Siewens wanted to make sex workers’ voices heard in an era in which sex work resurfaced as a theme of public debate in the context of the HIV/AIDS crisis. Before HIV/AIDS, Danish politics and activism often ignored sex work, describing it as ‘the oldest trade in the world’ or ‘a necessary evil of modern society’.^106^ During this three-year period, Stå Sammen was not only an attempt at self-care for persons selling sex; it was also a social movement of sex workers engaged widely in Danish society, with politicians, the media, other activist groups, and the medical system.

A few Danish politicians, such as Mogensen and Kjærsgaard, had already pointed to sex work as the most dangerous phenomenon regarding the spread of HIV/AIDS, but their statements were not representative of Danish MPs as a whole. Neither medical nor political authorities defined sex workers as a so-called risk group, but the press, by contrast, played a significant part in producing and sustaining the narrative that sex workers were particularly at risk of HIV/AIDS. The press often described the sex worker as dangerous, because she could transmit the infection to the majority population by having unprotected sex with her customers, accentuating the role of the sex worker as ‘the aids spreader’, ignoring the sex buyer’s role. In 1989, for example, when the newspaper Jyllands-Posten wrote that sex workers were unaffected by the information campaigns, and that they were spreading AIDS.^107^ Another example of a typical headline was ‘Panic in Copenhagen: Nobody checks up on the aids-whores’.^108^

The attention of the press and of some politicians to HIV/AIDS spreading through sex work remained an undocumented fantasy, but it was also an unsurprising consequence of medical attention to how the disease developed internationally. In 1987, the UFL reported the percentage of transmission through drug use in the United States to have been ‘consequently between 15 and 20 since 1981’.^109^ By 1989, the number was reported to be 25 percent.^110^ In southern Europe, and in some northern European cities such as Edinburgh and Stockholm, drug use was reported to make up 50 percent of all HIV transmissions.^111^ After 1986, when an HIV-infection through IV drug use was first registered in Denmark, medical professionals expressed concerns that ‘in so far no preventive measures [were] initiated; an explosive development of HIV-infections among drug users must be expected’.^112^ Specifically concerning drug users also selling sex, Sand Petersen, warned that ‘with the large number of sex partners of these drug-addict prostitutes, one [could] fear, that a diffused and uncontrollable spread of HIV in the heterosexual population [could] take place’.^113^

In the wake of Sand Petersen’s article in *UFL*, screenings were made of data from two sexual health clinics, one in Aarhus and one in Copenhagen, but neither found any signs of explosive HIV-transmission among sex workers.^114^ In Copenhagen, only one woman of 556 tested individuals was found to be living with HIV, and the woman was registered both as a sex worker and as a drug user. The remaining 31 women, registered as having sold sex, all tested negative, as did the four male sex workers participating in the study.^115^ In 1989, Peter Ege published an article about HIV/AIDS and drug use, arguing that ‘it [was] remarkable that the prevalence [of HIV-infections] had not risen since 1986’, concluding that drug users had changed their behaviour and that ‘this change [was] impressive, considering how little help they [had] received’.^116^ In 1990, another group of doctors presented a similar argument, that ‘HIV-infected women appear[ed] to [have] change[d] their behaviour, when finding out they [had] HIV’.^117^

In the early 1990s, a few studies focused on HIV/AIDS among heterosexual drug users and sex workers. Smith et al. published an article in 1990, based on a study showing that 10 percent of all individuals, living with HIV in Denmark, were women, and that 44 percent of them were infected through IV drug use. The same study showed that HIV/AIDS was primarily found in and around Copenhagen. In 1992, a study confirmed the suspicion that HIV infections had spread among IV drug users in 1985-86 but had since been limited. It concluded that until 1991, the absolute number of AIDS cases among IV drug users in and around Copenhagen had been 27 men and 8 women; out of 40 men and 13 women in the country as a whole.^118^ In 1993, another study of the autopsies of 389 deceased IV drug users between 1988 and 1991 showed that out of 309 men, 34 had lived with HIV, a number that for women was 11 out of 80.^119^

Even if the intersections between selling sex and using drugs indicated an increased prevalence of HIV/AIDS among drug-using sex workers in Denmark, the Danish medical authorities never had any solid knowledge of its scope. In 1985 and in 1992, two medical studies screening women selling sex found no cases of HIV/AIDS among their participants, the 1992 study screening 212 women in Copenhagen and 53 in Aarhus.^120^ The complete absence of HIV/AIDS surprised even sex workers themselves, according to reactions published in *Stå Sammen.* ^121^ One anonymous woman wrote about the study that ‘I did not know, that it [was] the girls who should fear AIDS from the customers. I was not smarter than to think it was the other way around.’^122^ Reacting to a claim made by a well-known medical professional in the previous issue, another woman wrote that ‘about those girls who will lose the rubber for extra money, I believe you are wrong. We are so informed about diseases by now’.^123^ Jackie Siewens, the publishing editor, wrote that: ‘the result of the study shows very clearly that we know the rubber has been invented and that other people should learn from us … even though we are still blamed for the spread of HIV’.^124^ *Stå Sammen* quoted one of the medical professionals behind the study, Lene Nyvang, saying that the condom was also used in order to create a distance from the customer ‘but the primary reason for using a condom [was] in [her] opinion a sincere sense of responsibility in the face of the AIDS threat’. She added that ‘the customers know less about HIV than the women: all prostitutes have experienced custumers attempting to persuade them not to use a condom’.^125^

Armed with the confidence emerging from the results of the study, Jackie Siewens and the sex workers from Stå Sammen continued to press for changes, in particular the full legalization of sex work in Denmark. Siewens, for example, attended meetings with Minister of Social Affairs, Karen Jespersen, about the life conditions of sex workers.^126^ Sympathetic of Siewens’ arguments and activities, the Minister assisted the group’s funding, and she gave Siewens her typewriter, as her old one had broken down. Stå Sammen also connected with the youth branch of the Conservative Party in Copenhagen. Over a longer period, young conservatives and sex workers protested for the legalization of sex work, the unlikely alliance drawing welcomed media attention to the cause. Eventually, sex work was legalized in Denmark in 1999, four years after Stå Sammen discontinued their activities. Siewens and Stå Sammen did not feature in the parliamentary discussions or motivations for the new law. Yet, many of the arguments and ambiguities first formulated and brought to political attention did.^127^

Despite the characterization of men selling sex as the most dangerous of all groups, similar medical studies were never conducted with male sex workers. The question of whether HIV/AIDS would spread to the heterosexual population through the buying and selling of sex between men was, however, a pressing issue in both activist and political spheres. Anders Dahl and Steffen Jöncke, affiliated with the Aids hotlines and STOP AIDS, respectively, launched a study in 1987 of male sex workers.^128^ Upon its publication, the AIDS Secretariat in Copenhagen Council’s social office established a foundation and a centre for ‘Male Prostitution Social Work’.^129^ The new initiatives, headed by AIDS consultants in the council, Joel Fallov and Carsten Damm, aimed to prevent the spread of HIV/AIDS through male sex work. At the same time, however, the council stated explicitly that Jöncke and Dahl’s study had exposed how difficult the life conditions of male sex workers were, bringing to the council’s attention that male sex workers in Copenhagen needed public help and support. Fallov and Damm highlighted that the group needed both counselling and support for the necessary information to be effective.^130^

The city’s objectives were eventually formulated as ‘seeking to hinder the spread of HIV/AIDS as well as securing that HIV positive sex workers receive counselling and support’.^131^ Copenhagen Council later employed social workers, Eva Fibiger and Torben Bitsch, to map out the male sex work landscape in Copenhagen as well as inform about safer sex practices and offer counselling and assistance. The project, Prostitution af fyre (PAF), eventually ended in 1996 and the activities merged with other activities. Through the years that PAF operated in Copenhagen, Fibiger and her co-workers estimated being in touch with 150-200 men and boys.^132^ As the files and most of the documents related to the project were destroyed when the project ended,^133^ the only surviving and accessible documents from PAF consist of quarterly activity reports and briefs, as well as correspondence with LBL, one of PAF’s stakeholders.

Although HIV/AIDS was the conduit, through which attention to male sex work reached political and activist arenas, the male sex workers’ social and personal problems other than HIV/AIDS became PAF’s primary concern.^134^ PAF quickly began reporting that they could not teach the male sex workers much about safer sex practices, although, according to the reports, the men and boys appreciated being able to discuss their concerns about HIV/AIDS with the PAF project and gain access to free condoms and lubricants.^135^ At the same time, the project gained insights into the lives of a group of people, whose problems not only consisted of high-frequency sexual relations and fears of contracting HIV but also of substance use and homelessness, amounting to suicide attempts and recurrent hospitalizations.^136^ Fibiger and her co-workers used different strategies in order to establish contact with men and boys selling sex in Copenhagen. One of their core activities was to walk the streets in the evening and at night, in order to locate and approach the men and boys in the scene. They focused primarily on street prostitution around the City Hall Square, the Central Train Station, a handful of gay bars in the city centre, a gaming arcade in a central shopping mall, and a cinema close by. The surviving PAF files only mention contact with one male sex worker, living with HIV/AIDS, but oral sources suggest that HIV/AIDS was widespread among male sex workers.^137^ It was evident, however, that the individuals PAF engaged with needed the social services not only because of HIV/AIDS but for other reasons too. In 1994, one of the PAF social workers expressed this himself upon returning from an international conference in Edinburgh on sex work. Frustrated with the fact that the topic of health and sex work always amounted to HIV/AIDS, he wrote: ‘it is clear that a number of prostitution projects utilize the fear of HIV/AIDS in order to secure funds for other projects – without there being any documentation that prostitution constitutes any particular risk of infection – more the opposite’.^138^

## Approaching the end of the crisis

By the mid-1990s, the HIV/AIDS crisis began to lose its momentum, if not in actual transmissions then in its signification as a ticking bomb under the general population. This development eventually resulted in an increasing invisibility of HIV/AIDS, and in the notion of the heterosexual epidemic diminishing. This also led to a waning focus on the homosexual epidemic, though paradoxically the mid-1990s were the years when both infection and death rates among gay men peaked. In the same way that large-scale attention to the homosexual epidemic had been contingent with the question of whether the disease could spread heterosexually ten years earlier in the mid-1980s, the fact that it did not really do so, at least not epidemically, meant that the homosexual focus disappeared again.

At the beginning of the crisis, LBL argued for HIV/AIDS to be understood as not (only) a homosexual problem. By the 1990s, the gay activists eventually took issue with the waning homosexual focus. One issue was about financing activities. As the STOP AIDS campaign was not permanently funded, LBL had to negotiate its budget with the State every year. Furthermore, LBL itself did not receive any public funding for its regular activities, despite the fact that LBL mobilized its volunteer activists to carry out many of the campaigns, such as condom distributions as well as information meetings and events. Another issue was that LBL’s best argument no longer seemed as urgent: Preventing the heterosexual epidemic was no longer an end, to which the investment in a strong and attractive gay community, in the shape of LBL, was a logical means. From the late 1980s onwards, homosexual HIV infection rates stagnated and the rates of other venereal diseases plummeted already from 1986 to 1987.^139^ This development was a product of LBL’s success, of course, but it also deflated the sense of urgency about the homosexual epidemic. Whereas the authorities had been willing to adopt the interests of LBL when the crisis was in its early stages, the association’s interests did not receive the same attention some years in. When LBL argued for the importance of destigmatizing gay sex and practices such as cruising, for example, the authorities did not offer any support. The political authorities were also oblivious to LBL’s other efforts in the early 1990s of ‘Regaying HIV’,^140^ which meant to lobby for the return of medical and political foci to the overwhelming majority of homosexual victims. Meanwhile, the politicians did offer other concessions to gays and lesbians. In 1987, homosexuality was included in anti-discrimination bill, and in 1989, the parliament famously passed the same-sex registered partnership bill. Both legal changes were considered significant improvements in homosexual rights in Denmark, and LBL welcomed and celebrated them. Yet, the ambitions of fundamentally challenging the norms for sexual relationships and gender roles also held by some LBL activists were compromised by the integrationist measures.

LBL’s influence on official Danish responses to HIV/AIDS ended in 1994, after a decade of engagement. As the association faced financial difficulties, the authorities demanded that the STOP AIDS campaign be removed from its influence along with the national telephone hotlines.^141^ Gay activist initiatives continued in STOP AIDS and elsewhere, in particular in a patient group, Positivgruppen. The days of the alignment and cooperation, however, between the largest gay and lesbian association and the political and medical authorities were over, and so was the special attention of the authorities to other gay activist aims and aspirations. The first large STOP AIDS initiative after the separation between the campaign and LBL, an information campaign explicitly ‘re-gaying HIV’ by drawing attention to the risk of infection through anal sex, was heavily criticized by the just-resigned director of the National Health Authority, Palle Juul-Jensen. In a book published in 1995, Juul-Jensen not only criticized the explicit nature of the information campaign, but he also wrote that the entire cooperation with LBL was a mistake, as it had framed ‘natural’ heterosexuality and ‘abnormal’ homosexuality as equals.^142^ Resonating more clearly with the idea of preventing the heterosexual epidemic, STOP AIDS eventually redirected its focus towards the ‘unemancipated’, or ‘closeted’ gay man and the bisexual, applying the new terminology, men having sex with other men (MSM).^143^ By focusing also on MSM rather than gay men only, STOP AIDS could hold onto its relevance and funding, at least for some time.

Exactly at the same time as the Danish Health Authority discontinued its cooperation with LBL, another development illustrated how resolute HIV/AIDS responses could still be when they directly aligned with the notion of preventing the heterosexual epidemic. In the wake of a criminal trial against a Danish national of Haitian origin, parliament approved adding ‘exposing others to infection with a deadly disease’ to the penal code.^144^ The trial had acquitted the man of charges made by former lovers, to whom he had not disclosed his HIV positive status. This led to a subsequent press campaign against the man and a speedy legal process in Parliament. The addition to the penal code of exposing others to infections with a life-threatening disease was a rupture in the Danish political responses to HIV/AIDS. The penal code compromised the principles of anonymity and voluntariness and it led to increasing stigma. The new penal code indicated that responses, ensuring anonymity and voluntariness, were suitable as long as they mostly concerned minoritized and marginalized communities, but when the heterosexual population was at risk, they no longer sufficed. When a heterosexual (black) man risked the lives of his female (white) lovers, political responses needed to intensify.^145^ While none of the acquitted man’s former lovers contracted the virus, the press campaign against him and the resolute political intervention showed how any potential route to a heterosexual epidemic needed to be aborted immediately and harshly.

This was true not only in the press and in politics, but also among medical professionals. In the 1990s, articles in UFL about HIV/AIDS were increasingly about potential avenues of heterosexual infections, in particular the so-called vertical infections, meaning passing of the virus from mother to child. Women with HIV/AIDS in Denmark were always an absolute minority. Nevertheless, the authorities continuously feared that the percentage of women with HIV/AIDS would increase. In 1989, it was known that out of the 494 people in Denmark who had been diagnosed with AIDS in total, 26 of them were women.^146^ In 1990, it was known that 115 Copenhagen women had tested positive for HIV in the period 1985–1988, and that 55 percent of them were also drug users.^147^ Regarding infection among pregnant women, the figures in Denmark were more uncertain. In 1989, a large study was conducted in Aarhus to gain insight into the extent of the problem. Data from ninety-five percent of all births in the region, Denmark’s second-most populous, were included in the study over a period of three months, but no infections were found.^148^ In neighbouring Sweden, where HIV tests had been offered systematically to pregnant women since 1986, 52 infections had been detected out of just over 424,000 tests over eight years.^149^ By the end of 1992, it was known that a total of 21 infants in Denmark had been infected with HIV up to that point. Of these, 12 had developed AIDS, and six children had already died.^150^ In comparison, in Edinburgh, Scotland, known as the ‘Aids capital of Europe’, the city accounted for over 30 percent of the total number of women with HIV in the UK, and by 1993, 500 children in the city were known to have care needs as a consequence of the disease.^151^

The intensity of the Danish medical community’s attention to potential heterosexual diffusion of HIV never matched the epidemic’s actual development. Repeatedly, UFL reported that medical professionals did not believe that Denmark was facing a heterosexual epidemic.^152^ But the prospect of it was what guided new studies and initiatives. Eventually, such initiatives stagnated in the mid-1990s, around the time when medical professionals also discovered that treating patients with three different anti-viral substances significantly reduced virus levels and symptoms in AIDS patients. Jan Gerstoft and Court Petersen wrote in UFL in 1997, that ‘for the first time, medical professionals now [believed] that a lasting improvement through anti-viral treatment [was] possible’.^153^ The test results showed that combining three anti-viral components in the same patients was 10 to 40 times more effective than previous treatments with two components. Gerstoft and Petersen presented the idea of ‘the chronic HIV infection’:^154^ that with the right combination of anti-viral treatment, patients might one day live with the HIV virus without ever developing AIDS.

## Conclusion

This article has shown that to prevent the heterosexual epidemic was the over-arching notion, catalyzing responses to HIV/AIDS in Denmark. This was the case from the beginning of the epidemic in 1981 until the signification of it changed by the end of the millennium, when the idea of HIV without AIDS materialized. It applied widely and across the board: in HIV/AIDS politics, in activism, and in medical professional discussions. Stated bluntly: the more the disease appeared to constitute a threat to the heterosexual population, the higher the amount of political attention, response, and intervention. The more an activist response appeared to align with the effort of preventing the heterosexual epidemic, the more urgent and important the response appeared. The more the virus encroached on the heterosexual population, the more medical professionals were alarmed. The significance of this analysis is that what appeared to be a very heterogeneous set of responses to the disease in Denmark was actually related and rooted in the same objective. This objective, moreover, was not directed by statistics of the actual spread of the disease.

That the notion of preventing the heterosexual epidemic leveraged Danish responses to the epidemic notably did not mean that individual heterosexual victims of the disease received the most attention, care, and support. Nor did it mean that other groups, to whom the virus and disease provided a much more substantial threat and lived reality, were ignored. On the contrary, heterosexual victims of HIV/AIDS in Denmark who were not haemophiliacs or using drugs were very unlikely victims, and often, invisible ones too. Many kept their status and disease a secret and faced huge stigma and loneliness. At the same time, some of the marginalized and minoritized groups hit hardest by HIV/AIDS were able to align existing and or new objectives with preventing the heterosexual epidemic, and see such objectives materialize as life-improving realities.

Gay activists and organizations were instrumental in formulating the official political policy towards HIV/AIDS. Their voices were heard, and their demands were accommodated because they aligned from the very beginning with the idea of contributing to preventing the heterosexual epidemic. Recognized for taking responsibility in the face of the crisis, gays and lesbians were awarded political concessions that – perhaps were on their way anyway – but perhaps became realities more rapidly in the face of the epidemic. When the epidemic appeared to be stagnating among MSM and not spreading to the general population from ‘closeted gay’ or ‘bisexual men’, however, the attention to the group’s heavy burdens with HIV/AIDS waned. This happened despite the fact that well into the new millennium, HIV/AIDS continued to hit the Danish gay male population and community disproportionately hard, although public spending and attention decreased continuously. The collective ban on donating blood to all men ever to have had any sexual relation with another man remained enforced well into the 2020s.

The haemophiliac community in Denmark was also hit disproportionately by HIV/AIDS. Ninety-one individuals with haemophilia, out of about 300 in the country in total, contracted the virus. Roughly two-thirds of them died of AIDS-related conditions. Still almost exclusively framed as a gay disease in 1985, it took a transfusion transmission of the virus to a young mother before political attention to the haemophiliac risk gained attention. Only when blood-borne infection appeared to happen to a member of the public were haemophiliac activists able to raise awareness of their concerns. Haemophiliacs were never framed as a threat to the general population, rather as innocent victims of the epidemic. This translated to an ambiguous visibility. On the one hand, haemophiliacs struggled to have their perspectives and real-life problems heard as these did not connect directly with preventing the heterosexual epidemic. On the other hand, haemophilic victims of HIV/AIDS faced no blaming stigma as a group – even if individual experiences of living with HIV/AIDS were far from stigma free.

Besides gay men, MSMs, and haemophiliacs, three other groups were disproportionally affected by or associated with HIV/AIDS in Denmark. These groups were IV drug users, sex workers of all genders, and non-white migrants or exiles in Denmark. All these groups, as well as individuals to whom more than one affiliation applied, were heavily affected by the notion of preventing the heterosexual epidemic. Blood-borne infections through IV drug use were a massive concern for political and medical professionals alike from the mid-1980s onwards. IV drug users were unambiguously identified as dangerous and potential spreaders of the disease. Such attention stigmatized an already heavy marginalized group. At the same time, however, treatment policies towards drug users also changed to some degree towards more inclusion and appreciation of the individual and their needs.

In some cases, when sex work and drug use intersected, anxiety about diffusion of the virus in the heterosexual population only accelerated. Drug using or not, female sex workers were consistently pointed out by the Danish press as potential ‘infection bombs’ and as ‘jeopardising the lives of Danish family fathers’. Such women were seen as agents of the epidemic, never as victims, and in reality, often, they were not. Although there was considerable medical, political, and activist attention directed at sex workers and their role in the epidemic, the large-scale studies of virus diffusion among sex workers never identified any prevalence. Solely among drug-using women who also sold sex, and among male and trans sex workers, were HIV/AIDS rates found to be higher than in the general population.

The attention to sex work that derived from preventing the heterosexual epidemic resulted in a variety of responses and contingencies. The epidemic stigmatized all sex workers even more than they already were. At the same time, however, the poor life conditions of many sex workers of all genders came to the fore. Such conditions became political topics, and several laws and practices were eventually changed. In addition, as it was the case with the gay and lesbian community, policy makers and politicians also consulted a community of sex workers. They, too, aligned existing agendas with the notion of preventing the heterosexual epidemic: the agenda of decriminalizing sex work in Denmark completely. Although this did not happen until 1999, and HIV/AIDS was never mentioned as a motivation for it, all the initiatives that had arisen as a consequence of the HIV/AIDS crisis were referred to.

The level of political response and intervention that preventing the heterosexual epidemic could leverage was illustrated most powerfully by the changing of the penal code in 1994. In the aftermaths of an extensive moral panic about a (black) heterosexual man’s sexual encounters with (white) heterosexual women, Danish politicians deferred unequivocally from the principles they had defined themselves in similar explicit terms seven years earlier. When the risk of diffusion came so close to the heterosexual public as the infamous case seemed to suggest, calls for action could not be ignored. As the medical treatment breakthrough came by the mid-1990s, and the heterosexual epidemic was established prevented by the end of the decade, responses to HIV/AIDS in Denmark faded quickly. By the turn of the millennium, HIV/AIDS seemed to be a problem overcome. Conversations about the disease turned to whether responses to HIV/AIDS had actually scared the heterosexual population unnecessarily. Of course, the success of preventing a heterosexual epidemic was as invisible an achievement as it had been in guiding and dominating discourse throughout the crisis.

## Footnotes

1

Deborah Gould , Moving Politics. Emotion and ACT UP’s Fight against AIDS (Chicago: Chicago University Press, 2009); Douglas Crimp , Melancholia and Moralism: Essays on AIDS and Queer Politics (Cambridge: MIT Press, 2002).

2

Christian W. McMillen , Pandemics: A Very Short Introduction (New York: Oxford University Press, 2016), 2–5 .

3

David Arnold et al., ‘The Pandemic and History’, The American Historical Review, 127, 3 (2022), 1340–1378, 1345.

4

Jörg Vögele , Luisa Rittershaus , and Katharina Schuler , ‘Epidemics and Pandemics – the Historical Perspective. *Introduction*’, Historical Social Research Supplement, 33 (2021), 7–33, 8 .

5

Signild Vallgårda , ‘Problematizations and Path Dependency: HIV/AIDS Policies in Denmark and Sweden’, Medical History, 51, 1 (2007), 99–112 17200699 10.1017/s0025727300000910PMC1712379.

6

Erik Albæk , ‘Denmark: AIDS and the Political “Pink Triangle”’, in David L. Kirp and Ronald Bayer (eds.), AIDS in the Industrialized Democracies (New Brunswick: Rutgers University Press, 1992), 282–283 .

7

Camilla Bruun Eriksen and Michael Nebeling Petersen , ‘The Racialisation of AIDS in Denmark: Tabloid Media Scandals of Sex, Race and Contagion’, European Journal of Cultural Studies, 28,3 (2024), 904–919. 10.1177/13675494241289317 (Original work published 2025).

8

Paula A. Treichler , How to Have Theory in an Epidemic: Cultural Chronicles of Aids (Durham: Duke University Press, 1999), 11 .

9
*Ibid.*

10
*Ibid.*

11

Jan Gerstoft , ‘Erhvervet immundefekt hos homoseksuelle mænd: Et nyt sygdomsbillede’, Ugeskrift for læger, 11 (1982), 771–776 7101503.

12 FK, ‘Livsfarlig kræft blandt homoseksuelle’, *Jyllands-Posten* (15.03.1982), 2.

13 FK, ‘Homoseksuelle og narkomaner sniffer sig til livsfarlig kræft’, *Jyllands-Posten* (09.10.1982), 16.

14

Per Michaelsen , ‘Hver 3. bøsse aids-smittet’, Ekstra Bladet (14.02.1985) 1,6 .

15

Per Michaelsen , ‘Bøsse-miljøet trodser AIDS-frygten: Vi boller bare videre’, Ekstra Bladet (18.02.1985), 6 .

16 Henning Jørgensen, ’AIDS – myter, skræmmemiddel og homopolitik, *PAN*, 4 (1983), 2.

17 See Tobias de Fønss Wung-Sung , ‘Radical Means with Compliant Ends: Transformations of the Gay and Lesbian Movement in Denmark between Stonewall and The Aids Crisis’, in Knud Andresen , Sebastian Justke , Stefanie Schüler-Springorum (eds.), Vom Ich zum Wir und wieder zurück? Subjektverständnisse zwischen Politisierung und Entradikalisierung seit den 1960er (Göttingen: Wallstein Verlag, 2023), 62–80 .

18
*Ibid.*

19
*Ibid.*

20

Craig Griffiths , The Ambivalence of Gay Liberation: Male Homosexual Politics in 1970s West Germany (Oxford: Oxford University Press, 2021).

21

Peter Edelberg , ‘From Criminal Radicalism to Gay and Lesbian Lobbyism: A Transnational Approach to the Scandinavian Homophile Movement, 1948–1971’, Scandinavian Journal of History, 49, 4 (2024), 513–536. 10.1080/03468755.2024.2336967 and Peter Edelberg , ‘Revolutionaries and Reformists: A Transnational Perspective on the Scandinavian LGBT Movement, 1972–2022’, Scandinavian Journal of History, 50, 3 (2024), 312–336. 10.1080/03468755.2024.2430992.

22 Hansen, Bent og Steffen Jensen, Henning Sandau og Kurt Sandau: Den lyserøde trekant mellem politik, aids og kultur: Erindringer fra Landsforeningen for Bøsser og Lesbiske fra 1971 til 2002 og lidt mere, (København: BoD: 2020).

23 Bech, Henning og Karin Lützen. *Lyst Eller Nød?: Kvinders og mænds homoseksualitet: Redegørelse fra Kommissionen til Belysning af homoseksuelles situation i samfundet* (København: Kommissionen til belysning af homoseksuelles situation i samfundet, 1986).

24

Robert J. Biggar et al., ‘Immunsuppressionssyndromet hos homoseksuelle mænd’, Ugeskrift for læger, 11 (1982), 777–780 7101504.

25
*Ibid.*

26
*Ibid.* and Gerstoft, *op. cit.* (note 11).

27

Niels Strandberg Pedersen , ‘Epidemic Kaposi’s Sarkoma and Opportunistic Infections in Homosexual Men’, Ugeskrift for læger, 29 (1983), 2232–2234 .

28

Jan Gerstoft , ‘Infections of Homosexual Men and the Acquired Immunodeficiency Syndrome’, Ugeskrift for læger, 4 (1983) 252–254 .

29
*Ibid.*

30 Pedersen, *op. cit.* (note 27).

31
*Ibid.*

32
*Ibid.*

33

Viggo Faber , ‘Helbredsundersøgelser for AIDS, PRE-AIDS og mistanke om AIDS’, Ugeskrift for læger, 36 (1984), 2712–2714 .

34

Jens O. Nielsen , ‘AIDS – Status 1984’, Ugeskrift for læger, 5 (1984), 367–368 .

35 Danish National Archives (DNA): LBL 173: ‘Aids-udvalget 1.11-12. Korrespondence, 1984’, ‘Gay Health Congress, Presentations, Denmark, Kopenhagen [sic.] by Tjerk v.d. Berg’.

36

Tjerk van den Berg , ‘Syfilis er i byen – nu kontrol i saunaerne’, PAN, 3 (1982), 19 .

37

Peter Kryger , et al., ‘Hepatitis blandt homoseksuelle i to københavnske saunaer’, Ugeskrift for læger, 17 (1984), 1276–1279 6474587.

38 LBL 173, *op cit.* (note 35).

39 DNA: LBL 173 ‘Aids-udvalget 1.11-11. Rapport fra tjenesterejse til Århus, 19.-21. oktober 1983. Epidemiologisk Afd. Statens Seruminstitut’.

40
*Ibid.*, ‘Landsformand Henning Jørgensens tale ved Aids-konference i Århus fredag den 21. oktober 1983.’

41 DNA: LBL 173: ‘Aids-Udvalget 1.11.-7. Møder på Rigshospitalet, 13/8 og 1/10 1984’.

42 Folketingstidende 1983–1984, 2. samling, 1051–52.

43
*Ibid.*

44
*Ibid.*

45

Gunhild Lange Wantzin , ‘AIDS og Human T-celleleukemi/lymfomvirus-lll i Danmark’, Ugeskrift for læger, 5 (1985), 389–391 2983469.

46

Maiken Arendrup et al., ‘LAV/HTLV-lll-antistof hos patienter med akut hepatitis B i årene 1975–1984,’ Ugeskrift for Læger, 14 (1986), 825–828.3010525

47 Wantzin, *op. cit.* (note 45).

48

Henning Jørgensen , ‘AIDS’, PAN, 2 (1985), 2, 5–6 .

49 DNA: LBL 175: ‘Sundhedsgruppen 1.12.-8. Høring om AIDS, Rigshospitalet, 30/10 1985’.

50 DNA: LBL 175: ‘Sundhedsgruppen 1.12.-8. Indenrigsministeriets debatmøde om AIDS, 7/11 1986’, ‘Jesper Jarmbæk Indlæg’.

51

Terkel Andersen , ‘AIDS – undersøgelse’, Medlemsblad Danmarks Bløderforening, 2–3 (1983), 9 .

52

Johannes Gormsen , ‘AIDS’, Medlemsblad Danmarks Bløderforening, 4 (1983), 7–10 .

53 Søren Brask, ‘Aids og hæmofili’, Medlemsblad Danmarks Bløderforening, 3–4 (1984), 3–7.

54
*Ibid.*

55
*Ibid.*

56

Jørgen Madsen (ed.), Danmarks Bløderforenings jubilæumsskrift *, 1970–1995* (Copenhagen: Antenneforeningen Kolibri, 1996), 15

57

Terkel Andersen , ‘Beretning 1985: Et hektisk og alvorligt år’, Medlemsblad Danmarks Bløderforening, 2 (1986), 10–16 .

58
*Ibid.*

59

Henrik Denta , ‘Erstatning til Aids-ramte’, Medlemsblad Danmarks Bløderforening, 3 (1986), 6 .

60

Henrik Bach , ‘Lille dreng død af AIDS – blev smittet med inficeret blod under transfusion i USA’, Politiken (13.04.1985), 1 .

61

Anja Westphal , ‘AIDS alarm: 10.000 bærer smitte’, Politiken (04.06.1985), 1 .

62 Anders-Peter Mathisen, ‘80 får AIDS inden nytår’ *Politiken* (03.06.1985), 2.

63

Tobias de Fønss Wung-Sung , ‘Aids angår alle’, TEMP, 28, (2024), 191–192 .

64
*Ibid.*

65
*Ibid.*

66
*Folketingstidende 1985-86*, forhandlingerne, 1787–1788, 2206, 2215, 2220–2222.

67 DNA: LBL 178: ‘Aids-aktionsudvalget 1.13-11. Spørgsmål fra Folketingets kommunaludvalg samt svar fra Indenrigsministeren 1984’.

68
*Ibid.*

69
*Folketingstidende 1985–86*, lovforslag, L 57, 1769–1772. https://www.folketingstidende.dk/samling/19851/lovforslag/l57/index.htm and *Folketingstidende 1987-88 1. samling*, lovforslag, L 11, 207–210. https://www.folketingstidende.dk/samling/19871/lovforslag/l11/index.htm.

70 Andersen, *op cit.* (note 57); Bruno Pedersen, ‘Lov er lov – og den skal afskaffes’, *PAN*, 7 (1987), 2.

71
*Folketingstidende 1987-88 1. samling*, lovforslag, L 157, 3151–3160. https://www.folketingstidende.dk/samling/19871/lovforslag/l157/index.htm.

72

Michael Nebeling Petersen and Astrid Helene Nielsen , ‘Danske Aids-Kampagners Repræsentation af HIV/AIDS 1983–1996’, Kvinder, Køn & Forskning, 1 (2023), 80–101, 86–87.

73
*Ibid.*

74
*Ibid.*

75

Jens Ole Nielsen , ‘Hvad kan man kræve af AIDS-forskningen? Ugeskrift for læger, 14 (1986), 831 .

76

Gorm Lisby et al., ‘Erhvervet immundefekt-syndrom – AIDS’, Ugeskrift for læger, 14 (1986) 860–862 3705228.

77 Wantzin, *op. cit.* (note 45).

78

Maiken Arendrup et al., ‘LAV/HTLV-lll-antistof hos patienter med akut hepatitis B i årene 1975–1984’, Ugeskrift for læger, 14 (1986), 825–828 3010525.

79 Petersen, *op. cit.* (note 74).

80 DNA: LBL 181: ‘Indenrigsministeriets arbejdsgruppe vedrørende generel oplysning om Aids’, ‘Redegørelse om planlægningen af den fremtidige informationsindsats over for sygdommen AIDS’ (14.10.1985).

81 DNA: LBL 180: ‘Amtsrådsforeningen’, ‘Internt notet vedr. møde i Amtsrådsforeningen’ (05.11.1985).

82 DNA: LBL 175: ‘Aidsaktionsudvalget 1.13.-7. Møde mellem landforeningen og indenrigsministeren, 9. juni 1986’, ‘Referat af mødet’ (09.06.1986).

83 Vallgårda, *op. cit.* (note 5), For the most comprehensive study of the HIV/AIDS Crisis in Sweden, see David Thorsén, ‘Den Svenska Aidsepidemien: Ankomst, Bemötande, Innebröd’. Doctoral Thesis (Uppsala: Acta Universitatis Upsaliensis, 2013). See also Lennerhed, Lena , og Jens Rydström , ‘Professional Activism: AIDS and Civil Society in Sweden, 1982–2000’, Kvinder, Køn & Forskning, 35, 1 (2023), 44–64 . https://doi.org/10.7146/kkf.v35i1.131935.

84 DNA: LBL 180: ‘Aftale: Landsforeningen for Bøsser og Lesbiske og Indenrigsministeriet’ (18.09.1986).

85
*Folketingstidende 1986-87, 1. samling*, forespørgsler, F 27, 9809–9810. https://www.folketingstidende.dk/samling/19861/forespoergsel/F27/19861_F27_M67_referat.pdf.

86
*Ibid.*, 9813, 9818, 9823, 9831.

87
*Ibid.* 9836–9839.

88
*Ibid.*

89
*Ibid.*, 9837.

90
*Ibid.*, 9844–9845.

91 Amtsrådsforeningen i Danmark og Alkohol-og Narkotikarådet, *Konference om stofmisbrugere og AIDS*, (København: Amtsrådsforeningen i Danmark, 1987)

92
*Ibid.*, Arne Grünfeld: *Sammenfatning, 7.*

93
*Ibid.*, 9–10.

94 See Esben Houborg and Olga Juul Eriksen , ‘Udviklingen i den faglige opfattelse af metadon fra 1977-2000’. Stof, 33 (2019). https://psy.au.dk/fileadmin/CRF/STOF/STOF_pdf_er/STOF_nr._33/08_STOF_nr._33_Udviklingen_i_den_faglige_opfattelse_af_metadon_fra_1977_til_2000.pdf.

95 Amtsrådsforeningen, *op. cit.* (note 90), 39–44.

96
*Ibid.*, 69.

97 Sundhedsstyrelsen AIDS-sekretariatet, ‘Omlægninger narkobehandlingen’, *AIDS NYT*, 5 (1987).

98 Sundhedsstyrelsen AIDS-sekretariatet, ‘Mere metadon til narkomaner’, *AIDS NYT*, 8 (1987).

99 Sundhedsstyrelsen, *op. cit.* (note 96).

100 Sundhedsstyrelsen AIDS-sekretariatet, ‘HIV-smittede kan komme i Metadonbehandling på kort tid’, *AIDS NYT*, 20 (1988).

101 Sundhedsstyrelsen AIDS-sekretariatet, ‘1 million sprøjter til Københavns Stofmisbrugere’, *AIDS NYT*, 30 (1989)

102

Graham Scambler , et al. ‘Women Prostitutes in the Aids Era.’ Sociology of Health and Illness,12, 3 (1990), 260–73.

103
*Ibid.*, 264.

104

Marion Pluskota , ‘Selling Sex in Amsterdam’, in Magaly Rodríguez García et al. (eds.), Selling Sex in the City: A Global History of Prostitution, 1600s-2000s (Leiden: Brill, 2017), 27–59 .

105 Susan P. Conner, ‘The Paradoxes and Contradictions of Prostitution in Paris’ in *Ibid.*, 195.

106

Ida Blom , ‘From Coercive Policies to Voluntary Initiatives’, Scandinavian Journal of History, 33, 1 (2008), 52–74 .

107

Jens Grund and Niels Ole Quist , ‘Usikker Sex’, Jyllands-Posten (29.10.1989), 6 .

108 KMH: ‘Panik i København: Ingen har check på AIDS-luderne’, *Ekstra Bladet* (17.09.1985), 15.

109

Court Petersen et al: ‘Sen optræden af HIV blandt danske IV stofmisbrugere’, Ugeskrift for læger, 44 (1987) 2963–2964 3433483.

110

Peter Ege , ‘Stofmisbrug og HIV-infektion’, Ugeskrift for læger, 10 (1989), 616–618 2922827.

111
*Ibid.*

112 Petersen, *op. cit.* (note 108).

113

Carsten Sand Petersen , ‘Forekomsten af antistof’, Ugeskrift for læger, 14 (1986), 821–223010523.

114

Jørgen Esmann et al., ‘HIV-Screening i venereaklinikkerne i Aarhus Amt’, Ugeskrift for læger, 5 (1988), 291–292 3388616. Anne-Marie Worm , ‘Hiv-overvågning’, Ugeskrift for læger, 17 (1989), 1063–642734870.

115
*Ibid.* Worm.

116 Ege, *op. cit.* (note 109).

117

Else Smith et al., ‘HIV-smittede kvinder i København’, Ugeskrift for læger, 48 (1990), 3603–3606 2256221.

118

John S. Andersen et al., ‘Udbredelsen af HIV-smitte og AIDS blandt stofmisbrugere’, Ugeskrift for læger, 35 (1992), 2368–2371 1413152.

119

Birgitte Kringsholm et al., ‘Forekomst af antistoffer hos stofmisbrugere obduceret i Kbh, 1988-91. (fork)’ *Ugeskrift for læger* , 17 (1993), 1294–1296 8506578.

120

Aids-sekretariatet, Sundhedsstyrelsen , ‘Ingen fund af HIV-smitte blandt 212 prostituerede’, AIDS NYT, 53 (1992), 3–8 .

121

N.N. , ‘Ingen fund af HIV-Smitte blandt 212 prostituerede’, Stå Sammen, 2 (1992), 25–29 .

122
*Ibid.*

123
*Ibid.*

124
*Ibid.*

125
*Ibid.*

126

Jackie Siewens , ‘Kære venner’, Vi står sammen, 8 (1993), 4 .

127
*Folketingstidende 1998-99*, lovforslag, L 43, 930–942. https://www.folketingstidende.dk/samling/19981/lovforslag/L43/19981_L43_som_fremsat.pdf.

128

Anders Dahl , Træk af den mandlige prostitution (København: Projektgruppen, 1990).

129 DAPHNE., m.fl. *Mandlig prostitution – en usynlighed: indlæg ved høringen om mandlig prostitution på Københavns Rådhus den 7. april 1992* (København: Socialpolitisk Forlag, 1992).

130

Joel Fallov , Socialt arbejde med mandlige prostituerede: projektrapport (København: Social-og Sundhedsforvaltningen i København, 1992).

131 DNA: LBL 37: ‘Fonden for virke mod mandlig prostitution 1.07’, ‘Baggrundsbeskrivelse’.

132
*Ibid.*, ‘Redegørelse for sept-nov 1993’.

133
*Ibid.*, ‘Memo d. 19/10-92.’

134
*Ibid.*, ‘Abejdsopgaver for PAF i nov-dec 1992’.

135
*Ibid.*

136 DNA: LBL 37: ‘Fonden for virke mod mandlig prostitution 1.07.’ ‘Div. oplæg og rapporter 1991–1996’.

137
*Ibid.*, ‘Gennemgang af cases, 21/6-21/9-1992’.

138
*Ibid.*, ‘Redegørelse jul-aug 1994’.

139

MB , ‘Drastisk fald – kønssygdomme afspejler seksualadfærd’, PAN, 7 (1987), 18 .

140 DNA: LBL 176: ‘Re-gaying HIV: First European Conference on HIV and Homosexuality Copenhagen 17–18 February 1990.’

141 STOP AIDS, Nyhedsbrev, maj 1994: https://rs.cms.hu-berlin.de/ehaa/pages/view.php?ref=261&iaccept=on.

142

Palle Juul-Jensen , I statens tjeneste: Sundhedspolitisk efterskrift (Højbjerg: Hovedland, 1996), 14–17 .

143

Steffen Jöhncke et al., Mere End Blot ‘Sikker Sex’: Evaluering af Stop Aids-Kampagnen (Kbh: PAN, 1993).

144
*Folketingstidende 1993–94, 1. samling*, lovforslag, L 255, 8773. https://www.folketingstidende.dk/samling/19931/lovforslag/l255/index.htm.

145 Eriksen, *op. cit.* (note 6).

146

Anne-Marie Worm and Birgit Kvinesdal , ‘HIV-overvågning’, Ugeskrift for læger, 17 (1989), 1063–1064 2734870.

147

Else Smith et al., ‘Heteroseksuel HIV-smitte i Danmark’, Ugeskrift for læger, 23 (1990), 1668–1670 2363209; Else Smith et al., ‘HIV-smittede kvinder i København’, Ugeskrift for læger, 48 (1990), 3603–3606 2256221.

148

Anders Foldspand and Morten Hedegaard , ‘Gravides accept af tilbud om HIV-antistoftest’, Ugeskrift for læger, 45 (1990), 3360–3364 2238230.

149
*Ibid.*

150

May J. Olofsson , ‘Børn i HIV-smittede familier’, Ugeskrift for læger, 44 (1992), 3092–3094 .

151

Hannah J. Elizabeth , ‘Recovering Mothers’ Experiences of HIV/AIDS Health Activism in Edinburgh, 1983–2000’, in J. Weston and H.J. Elizabeth (eds.), Histories of HIV/AIDS in Western Europe: New and Regional Perspectives, 1980–2019 (Manchester: Manchester University Press, 2022), 168 40106611.

152

Michael Diepeveen , ‘Status Over HIV-Epidemien’, Ugeskrift for læger, 24 (1992), 1730–1734 .

153

Court Pedersen and Jan Gerstoft , ‘Antiviral kombinationsbehandling mod HIV’, Ugeskrift for læger, 45 (1997), 6655–6659 9411978.

154
*Ibid.*

